# Editorial: Multiscale brain modelling

**DOI:** 10.3389/fncel.2026.1783885

**Published:** 2026-02-09

**Authors:** Egidio D'Angelo

**Affiliations:** Department of Brain and Behavioral Sciences, University of Pavia, Pavia, Italy

**Keywords:** Alzheimer's disease, functional connectivity, multiscale brain modeling, network oscillations, neuronal excitability

This Special Topic touches one of the hottest fields in neuroscience, i.e., the capacity to build models of the brain crossing multiple scales. Building models can be done in many ways and addressing many different issues, spanning from theories of neuronal excitation to mesoscale models of network activity, whole-brain functions, and behavior. These models are now made feasible by recent developments in informatics and big data science.

Addressing the multiscale brain organization is fundamental not only to understand its inherent mechanisms of function but also to answer neuropathological questions and promote the development of new technologies for AI and health. A review on the issue is presented here in the paper by Krejcar and Namazi and additional information can be found in D'Angelo and Jirsa (2022) and Wang et al. (2024). The Authors have then covered two main areas of research: theoretical models of neuronal excitability, network oscillations, and brain activity, and models applied to the study of Alzheimer's disease.

Galinsky and Frank address the wave nature of the action potentials proposing an alternative framework to the standard Hodgkin-Huxley model for the action potential in axons. This is based on the Author's theory of electric field wave propagation in anisotropic and inhomogeneous brain tissues and addresses the limitations of the Hodgkin-Huxley model, including its inability to explain extracellular spiking, efficient brain synchronization, saltatory conduction along myelinated axons, and various other observed coherent macroscopic brain electrical phenomena. Pieramico et al. show how Hidden Markov Models can be used to analyze time series of neural activity. The study demonstrates that Time-Delay Embedded Hidden Markov Models performs better than Gaussian models in accurately detecting brain states from synthetic phase-coupled interaction data. Finally, Ghosh et al. present general trainable networks of Hopf oscillators to model high-dimensional electroencephalogram (EEG) signals across different sleep stages. The model, once embedded with a hidden layer, can faithfully predict the empirical EEG representing a step toward constructing a large-scale, biologically inspired model of brain dynamics.

Two papers address Alzheimer's disease. Fadel et al. present a model of functional connectivity changes with learning and memory in a mouse model based on data obtained from mutant mice. The APP/PS1 mice showed a pattern of hyperconnectivity, including the Default Mode Network, after learning. Modeling revealed functional connections that support learning and memory performance. These models show potential for early disease detection by identifying connectivity patterns associated with cognitive decline and may provide a means to understand how FC translates into learning and memory performance. Moravveji et al. show a sensitivity analysis of a mathematical model of Alzheimer's disease progression to unveil causal pathways. The study presents the first local sensitivity analysis of a multiscale ODE-based model of Alzheimer's Disease (AD) and captures the multifactorial nature of AD incorporating neuronal, pathological, and inflammatory processes at the nano, micro and macro scales. This detailed framework enables realistic simulation of disease progression and identification of key biological parameters that influence system behavior. This analysis identifies the key drivers of disease progression across patient profiles, providing insight into targeted therapeutic strategies.
